# Supplementary material for: Identification of patients at risk for pancreatic cancer in a 3-year timeframe based on machine learning algorithms
Source: Sci Rep. 2025 Apr 5;15:11697. doi: 10.1038/s41598-025-89607-8 (PMC11972345; doi:10.1038/s41598-025-89607-8)

S1 Fig. The Receiver Operating Characteristics Curve (AUROC 0.701 [0.684, 0.718]) of Model 2 on the held-out validation set to predict new-onset pancreatic cancer in a 3-year follow-up.

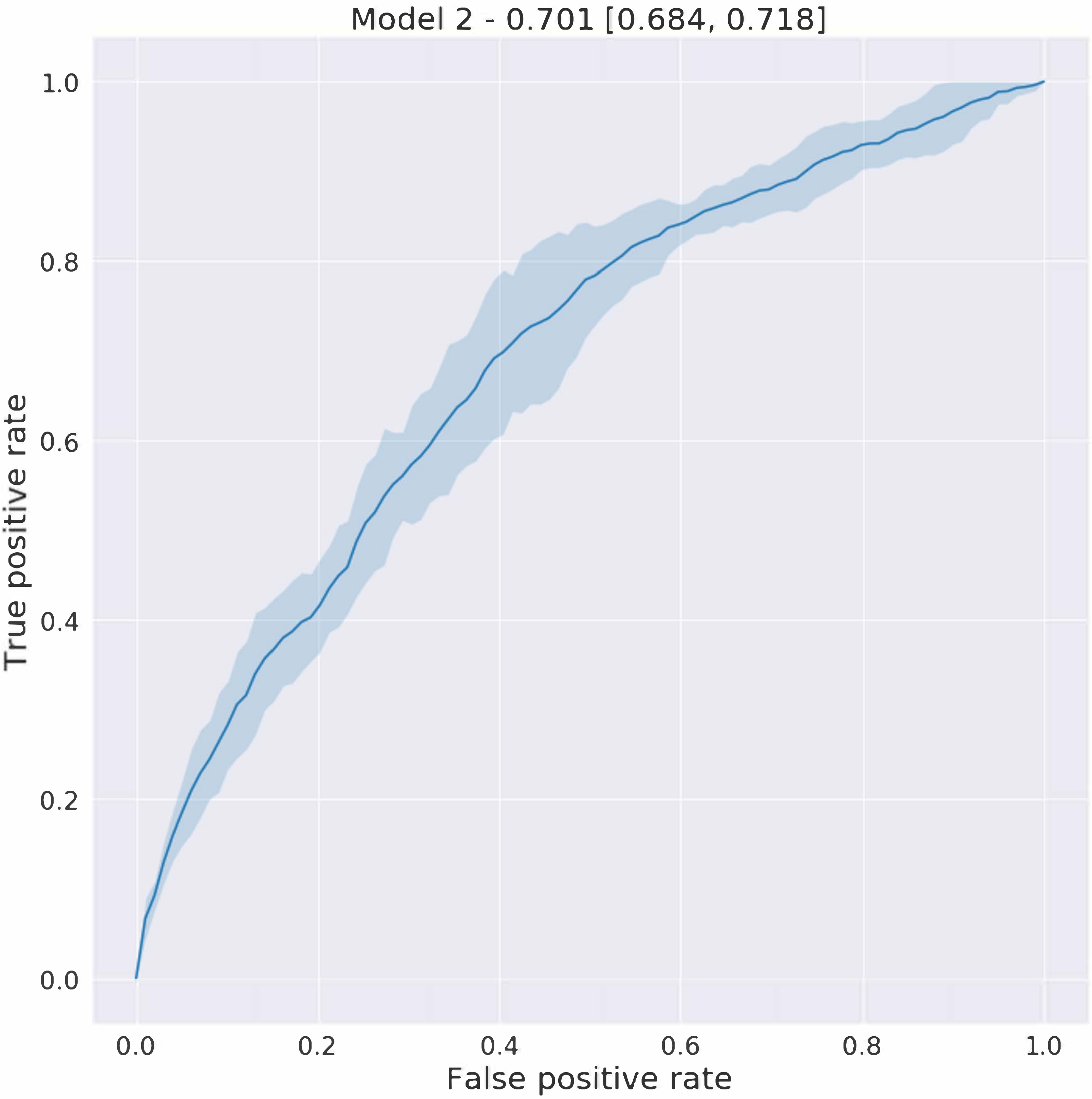

Supplement: Supplementary file 1 — Supplementary Information 1. [file 41598_2025_89607_MOESM1_ESM.pdf]
