# Supplementary material for: Identification of patients at risk for pancreatic cancer in a 3-year timeframe based on machine learning algorithms
Source: Sci Rep. 2025 Apr 5;15:11697. doi: 10.1038/s41598-025-89607-8 (PMC11972345; doi:10.1038/s41598-025-89607-8)
Supplement: Supplementary file 2 — Supplementary Information 2. [file 41598_2025_89607_MOESM2_ESM.docx]

**Supporting Information**

**S1 Fig. The Receiver Operating Characteristics Curve (AUROC 0.701 [0.684, 0.718]) of**

**Model 2 on the held-out validation set to predict new-onset pancreatic cancer in a 3-year follow-up.**

**S1 Table. Summary of settings and performance of related works on predicting pancreatic cancer with EHR data.**

**S2 Table. Definition of pancreatic cancers referring to ICD-10 codes**

| **ICD-10 Codes** | **Description** |
| --- | --- |
| C25.0 | Malignant neoplasm of head of pancreas |
| C25.1 | Malignant neoplasm of body of pancreas |
| C25.2 | Malignant neoplasm of tail of pancreas |
| C25.3 | Malignant neoplasm of pancreatic duct |
| C25.7 | Malignant neoplasm of other parts of pancreas |
| C25.8 | Malignant neoplasm of overlapping sites of pancreas |
| C25.9 | Malignant neoplasm of pancreas, unspecified |

**S3 Table. Normal ranges of lab values used in this study**

| **Lab** | **Threshold used for abnormal laboratory test** |
| --- | --- |
| A1C (Diabetes) | < 6.5% |
| CA19-9 | < 37 mg/dL |
| Lipase | < 234 U/L |
| Amylase | < 330 U/L |
| AST | < 40 |
| ALT | < 55 |
| *Albumin* | *< 3.4* |
| *Hemoglobin (HGB)* | *> 13 g/dL* |
| *Fasting glucose* | *< 126 mg/dL* |

*​*

**S4 Table. The full list of selected features by PheWAS. The unadjusted odds ratios on the case-control cohort and adjusted odds ratios of two models on the overall cohort are listed. *Note that the statistically significant features after bonferroni correction are marked (*** means corrected p-value < 0.001; ** means corrected p-value < 0.05; * means corrected p-value < 0.1)**

| **Diagnosis Features** | **Control (%)** | **Case (%)** | **P-value** | **Unadjusted Odds Ratio**  **[95% Confidence Interval]** |
| --- | --- | --- | --- | --- |
| Disease of pancreas; unspecified (ICD K86.9) | 2(0.02%) | 11(0.53%) | 5.61E-09 | 22.11 [4.90 ; 99.83] |
| Malignant neoplasm of transverse colon (ICD C18.4) | 1(0.01%) | 5(0.24%) | 1.05E-04 | 20.05 [2.34 ; 171.68] |
| Other chronic pancreatitis ((ICD K86.1) | 2(0.02%) | 8(0.38%) | 2.08E-06 | 16.06 [3.41 ; 75.67] |
| Hemangioma of intra-abdominal structures (ICD D18.03) | 1(0.01%) | 4(0.19%) | 7.94E-04 | 16.03 [1.79 ; 143.49] |
| Malignant neoplasm of unspecified fallopian tube (ICD C57.00) | 1(0.01%) | 4(0.19%) | 7.94E-04 | 16.03 [1.79 ; 143.49] |
| Cyst of pancreas (ICD K86.2) | 13(0.16%) | 46(2.20%) | 6.21E-29 | 14.45 [7.79 ; 26.80] |
| Peritoneal adhesions (postprocedural) (postinfection) (ICD K66.0) | 1(0.01%) | 3(0.14%) | 5.95E-03 | 12.02 [1.25 ; 115.58] |
| Neoplasm of uncertain behavior of digestive organ; unspecified (ICD D37.9) | 1(0.01%) | 3(0.14%) | 5.95E-03 | 12.02 [1.25 ; 115.58] |
| Encounter for breast reconstruction following mastectomy (ICD Z42.1) | 1(0.01%) | 3(0.14%) | 5.95E-03 | 12.02 [1.25 ; 115.58] |
| (ICD I48.21) | 1(0.01%) | 3(0.14%) | 5.95E-03 | 12.02 [1.25 ; 115.58] |
| Body mass index (BMI) 39.0-39.9; adult (ICD Z68.39) | 1(0.01%) | 3(0.14%) | 5.95E-03 | 12.02 [1.25 ; 115.58] |
| Presence of urogenital implants (ICD Z96.0) | 1(0.01%) | 3(0.14%) | 5.95E-03 | 12.02 [1.25 ; 115.58] |
| Personal history of poliomyelitis (ICD Z86.12) | 1(0.01%) | 3(0.14%) | 5.95E-03 | 12.02 [1.25 ; 115.58] |
| Chronic superficial gastritis without bleeding (ICD K29.30) | 1(0.01%) | 3(0.14%) | 5.95E-03 | 12.02 [1.25 ; 115.58] |
| Pseudocyst of pancreas (ICD K86.3) | 6(0.07%) | 16(0.77%) | 6.04E-10 | 10.74 [4.20 ; 27.48] |
| Hypertrophy of breast (ICD10 N62) | 4(0.05%) | 9(0.43%) | 8.98E-06 | 9.03 [2.78 ; 29.37] |
| Aftercare following surgery for neoplasm (ICD Z48.3) | 4(0.05%) | 9(0.43%) | 8.98E-06 | 9.03 [2.78 ; 29.37] |
| Neoplasm of unspecified behavior of digestive system (ICD D49.0) | 8(0.10%) | 16(0.77%) | 1.05E-08 | 8.05 [3.44 ; 18.84] |
| Personal history of other malignant neoplasm of kidney (ICD Z85.528) | 3(0.04%) | 6(0.29%) | 4.63E-04 | 8.02 [2.00 ; 32.10] |
| Acute frontal sinusitis; unspecified (ICD J01.10) | 2(0.02%) | 4(0.19%) | 4.26E-03 | 8.01 [1.47 ; 43.78] |
| Body mass index (BMI) 31.0-31.9; adult (ICD Z68.31) | 2(0.02%) | 4(0.19%) | 4.26E-03 | 8.01 [1.47 ; 43.78] |
| Personal history of other malignant neoplasm of rectum; rectosigmoid junction; and anus (ICD Z85.048) | 4(0.05%) | 7(0.33%) | 2.95E-04 | 7.02 [2.05 ; 24.00] |
| Incomplete uterovaginal prolapse (ICD N81.2) | 3(0.04%) | 5(0.24%) | 2.64E-03 | 6.68 [1.60 ; 27.98] |
| Hypertrophy of nasal turbinates (ICD J34.3) | 3(0.04%) | 5(0.24%) | 2.64E-03 | 6.68 [1.60 ; 27.98] |
| Malignant neoplasm of ascending colon (ICD C18.2) | 3(0.04%) | 5(0.24%) | 2.64E-03 | 6.68 [1.60 ; 27.98] |
| Other abnormal tumor markers (ICD R97.8) | 5(0.06%) | 8(0.38%) | 1.79E-04 | 6.42 [2.10 ; 19.65] |
| (ICD C20) | 4(0.05%) | 6(0.29%) | 1.56E-03 | 6.01 [1.70 ; 21.33] |
| Body mass index (BMI) 37.0-37.9; adult (ICD Z68.37) | 4(0.05%) | 6(0.29%) | 1.56E-03 | 6.01 [1.70 ; 21.33] |
| Other specified diseases of pancreas (ICD K86.89) | 11(0.13%) | 15(0.72%) | 1.50E-06 | 5.49 [2.52 ; 11.96] |
| Genetic susceptibility to other malignant neoplasm (ICD Z15.09) | 10(0.12%) | 13(0.62%) | 1.17E-05 | 5.23 [2.29 ; 11.94] |
| Benign lipomatous neoplasm of other sites (ICD D17.79) | 4(0.05%) | 5(0.24%) | 7.63E-03 | 5.01 [1.34 ; 18.67] |
| Encounter for surgical aftercare following surgery on the digestive system (ICD Z48.815) | 4(0.05%) | 5(0.24%) | 7.63E-03 | 5.01 [1.34 ; 18.67] |
| Genetic susceptibility to malignant neoplasm of breast (ICD Z15.01) | 9(0.11%) | 11(0.53%) | 8.97E-05 | 4.91 [2.03 ; 11.86] |
| Malignant neoplasm of unspecified ovary (ICD C56.9) | 6(0.07%) | 7(0.33%) | 2.27E-03 | 4.68 [1.57 ; 13.94] |
| Genetic susceptibility to malignant neoplasm of ovary (ICD Z15.02) | 8(0.10%) | 9(0.43%) | 6.78E-04 | 4.52 [1.74 ; 11.72] |
| Benign neoplasm of ascending colon (ICD D12.2) | 9(0.11%) | 9(0.43%) | 1.45E-03 | 4.01 [1.59 ; 10.12] |
| Acute pancreatitis without necrosis or infection; unspecified (ICD K85.90) | 7(0.08%) | 7(0.33%) | 4.98E-03 | 4.01 [1.41 ; 11.45] |
| Left upper quadrant pain (ICD R10.12) | 7(0.08%) | 7(0.33%) | 4.98E-03 | 4.01 [1.41 ; 11.45] |
| Other specified personal risk factors; not elsewhere classified (ICD Z91.89) | 27(0.32%) | 26(1.24%) | 1.15E-07 | 3.89 [2.26 ; 6.68] |
| Dependence on renal dialysis (ICD Z99.2) | 8(0.10%) | 7(0.33%) | 9.77E-03 | 3.51 [1.27 ; 9.69] |
| Personal history of other endocrine; nutritional and metabolic disease (ICD Z86.39) | 8(0.10%) | 7(0.33%) | 9.77E-03 | 3.51 [1.27 ; 9.69] |
| Thoracic aortic aneurysm; without rupture (ICD I71.2) | 13(0.16%) | 11(0.53%) | 1.54E-03 | 3.40 [1.52 ; 7.59] |
| Family history of malignant neoplasm; unspecified (ICD Z80.9) | 18(0.22%) | 15(0.72%) | 2.51E-04 | 3.35 [1.69 ; 6.66] |
| Primary osteoarthritis; unspecified hand (ICD M19.049) | 10(0.12%) | 8(0.38%) | 9.46E-03 | 3.21 [1.26 ; 8.14] |
| Other specified counseling (ICD Z71.89) | 23(0.27%) | 18(0.86%) | 1.26E-04 | 3.15 [1.70 ; 5.85] |
| Malignant neoplasm of unspecified kidney; except renal pelvis (ICD C64.9) | 14(0.17%) | 10(0.48%) | 7.89E-03 | 2.87 [1.27 ; 6.46] |
| (ICD R69) | 17(0.20%) | 12(0.57%) | 3.95E-03 | 2.83 [1.35 ; 5.94] |
| Long term (current) use of insulin (ICD Z79.4) | 43(0.51%) | 29(1.39%) | 1.59E-05 | 2.72 [1.70 ; 4.37] |
| Other specified diseases of liver (ICD K76.89) | 30(0.36%) | 20(0.96%) | 3.94E-04 | 2.68 [1.52 ; 4.73] |
| Cerebral infarction due to unspecified occlusion or stenosis of unspecified cerebral artery (ICD I63.50) | 24(0.29%) | 15(0.72%) | 3.88E-03 | 2.51 [1.31 ; 4.79] |
| Nausea (ICD R11.0) | 34(0.41%) | 21(1.00%) | 7.25E-04 | 2.49 [1.44 ; 4.29] |
| Family history of malignant neoplasm of other organs or systems (ICD Z80.8) | 21(0.25%) | 13(0.62%) | 7.75E-03 | 2.49 [1.24 ; 4.97] |
| Disease of stomach and duodenum; unspecified (ICD K31.9) | 28(0.33%) | 17(0.81%) | 2.81E-03 | 2.44 [1.33 ; 4.47] |
| Monoclonal gammopathy (ICD D47.2) | 24(0.29%) | 14(0.67%) | 9.31E-03 | 2.34 [1.21 ; 4.54] |
| Other obesity due to excess calories (ICD E66.09) | 24(0.29%) | 14(0.67%) | 9.31E-03 | 2.34 [1.21 ; 4.54] |
| Other diseases of stomach and duodenum (ICD K31.89) | 55(0.66%) | 31(1.48%) | 1.87E-04 | 2.27 [1.46 ; 3.54] |
| Overweight (ICD E66.3) | 43(0.51%) | 24(1.15%) | 1.16E-03 | 2.25 [1.36 ; 3.71] |
| Epigastric pain (ICD R10.13) | 72(0.86%) | 38(1.82%) | 1.26E-04 | 2.13 [1.43 ; 3.17] |
| Family history of malignant neoplasm of digestive organs (ICD Z80.0) | 63(0.75%) | 33(1.58%) | 4.04E-04 | 2.11 [1.38 ; 3.23] |
| Type 2 diabetes mellitus with hyperglycemia (ICD E11.65) | 56(0.67%) | 29(1.39%) | 1.09E-03 | 2.09 [1.33 ; 3.28] |
| Unspecified abdominal pain (ICD R10.9) | 144(1.72%) | 63(3.01%) | 1.50E-04 | 1.77 [1.31 ; 2.39] |
| Malignant neoplasm of unspecified site of unspecified female breast (ICD C50.919) | 101(1.21%) | 43(2.06%) | 2.89E-03 | 1.72 [1.20 ; 2.46] |
| Constipation; unspecified (ICD K59.00) | 111(1.33%) | 45(2.15%) | 5.39E-03 | 1.64 [1.15 ; 2.32] |
| Mixed hyperlipidemia (ICD E78.2) | 228(2.73%) | 89(4.26%) | 2.62E-04 | 1.59 [1.24 ; 2.04] |
| Type 2 diabetes mellitus without complications (ICD E11.9) | 459(5.49%) | 164(7.84%) | 4.71E-05 | 1.47 [1.22 ; 1.76] |

**S5 Table. The full list of intercept, coefficients, and p-values of the features in the logistic regression model. *Note that the statistically significant features after bonferroni correction are marked (*** means corrected p-value < 0.001; ** means corrected p-value < 0.05; * means corrected p-value < 0.1).**

| **Description** | **P-value** | **Adjusted odds Ratio** |
| --- | --- | --- |
|  |  | **[95% Confidence Interval]** |
| Intercept | 3.50E-01 | 0.811 [0.417, 1.206] |
| Other specified diseases of pancreas (ICD K86.89) | 0.00E+00 *** | 6.429 [4.734, 8.124] |
| Neoplasm of unspecified behavior of digestive system (ICD D49.0) | 6.99E-08 *** | 4.106 [2.243, 5.970] |
| Acute pancreatitis without necrosis or infection, unspecified (ICD K85.90) | 8.44E-14 *** | 4.019 [1.568, 6.470] |
| Malignant neoplasm of unspecified kidney, except renal pelvis (ICD C64.9) | 2.00E-15 *** | 3.947 [2.634, 5.259] |
| Age | 0E+00 *** | 2.924 [2.748, 3.100] |
| Primary osteoarthritis, unspecified hand (ICD M19.049) | 1.39E-10 *** | 2.868 [1.984, 3.752] |
| Family history of malignant neoplasm, unspecified (ICD Z80.9) | 2.22E-16 *** | 2.768 [2.136, 3.399] |
| Genetic susceptibility to other malignant neoplasm (ICD Z15.09) | 0.00E+00 *** | 2.159 [0.597, 3.720] |
| Hemangioma of intra-abdominal structures (ICD D18.03) | 2.54E-02 | 1.987 [0.959, 3.015] |
| Cyst of pancreas (ICD K86.2) | 2.44E-15 *** | 1.924 [1.127, 2.720] |
| Glucose > 126.0 | 1.45 E-04 * | 1.910 [1.223, 2.596] |
| Malignant neoplasm of unspecified site of unspecified female breast (ICD C50.919) | 0.00E+00 *** | 1.880 [1.660, 2.100] |
| Type 2 diabetes mellitus without complications (ICD E11.9) | 0.00E+00 *** | 1.876 [1.749, 2.002] |
| Personal history of poliomyelitis (ICD Z86.12) | 5.34E-02 | 1.797 [0.996, 2.597] |
| Cerebral infarction due to unspecified occlusion or stenosis of unspecified cerebral artery (ICD I63.50) | 5.50E-03 | 1.780 [1.119, 2.442] |
| Genetic susceptibility to malignant neoplasm of breast (ICD Z15.01) | 1.40E-05 ** | 1.758 [0.714, 2.801] |
| Disease of stomach and duodenum, unspecified (ICD K31.9) | 1.18E-01 | 1.610 [0.671, 2.549] |
| Pseudocyst of pancreas (ICD K86.3) | 4.41E-02 | 1.511 [0.871, 2.151] |
| Other specified diseases of liver (ICD K76.89) | 8.65E-04 * | 1.485 [1.144, 1.826] |
| Personal history of other endocrine, nutritional and metabolic disease (ICD Z86.39) | 5.48E-02 | 1.459 [0.902, 2.015] |
| Malignant neoplasm of unspecified fallopian tube (ICD C57.00) | 4.87E-08 *** | 1.427 [1.241, 1.613] |
| Malignant neoplasm of rectum (ICD C20) | 2.70E-01 | 1.418 [0.613, 2.224] |
| Disease of pancreas, unspecified (ICD K86.9) | 7.97E-04 * | 1.415 [1.135, 1.696] |
| Aftercare following surgery for neoplasm (ICD Z48.3) | 7.09E-01 | 1.342 [0.217, 2.467] |
| Obesity (BMI > 30) | 9.39E-02 | 1.238 [0.945, 1.531] |
| Personal history of other malignant neoplasm of rectum, rectosigmoid junction, and anus (ICD Z85.048) | 7.05E-03 | 1.263 [1.051, 1.475] |
| Body mass index (BMI) 31.0-31.9, adult (ICD Z68.31) | 4.10E-01 | 1.212 [0.789, 1.635] |
| Hypertrophy of breast (ICD N62) | 5.01E-01 | 1.208 [0.616, 1.800] |
| Gender (Male) | 4.98E-02 | 1.194 [0.984, 1.404] |
| Epigastric pain (ICD R10.13) | 4.52E-01 | 1.187 [0.754, 1.619] |
| Family history of malignant neoplasm of digestive organs (ICD Z80.0) | 1.93E-01 | 1.183 [0.892, 1.474] |
| Other obesity due to excess calories (ICD E66.09) | 4.06E-02 | 1.155 [0.997, 1.314] |
| Lipase > 234.0 | 5.28E-01 | 1.111 [0.790, 1.431] |
| Overweight (BMI 24~30) | 6.10E-01 | 1.087 [0.779, 1.395] |
| Other specified personal risk factors, not elsewhere classified (ICD Z91.89) | 2.59E-01 | 1.083 [0.937, 1.229] |
| A1C > 6.5 | 4.76E-01 | 1.058 [0.905, 1.211] |
| Other diseases of stomach and duodenum (ICD K31.89) | 6.85E-01 | 1.056 [0.787, 1.325] |
| Other specified counseling (ICD Z71.89) | 5.84E-01 | 1.055 [0.858, 1.252] |
| Unspecified abdominal pain (ICD R10.9) | 9.89E-01 | 1.003 [0.819, 1.187] |
| Dependence on renal dialysis (ICD Z99.2) | 1.00E+00 | 1.000 [1.000, 1.000] |
| Malignant neoplasm of transverse colon (ICD C18.4) | 1.00E+00 | 1.000 [1.000, 1.000] |
| Carcinoid syndrome (ICD E34.0) | 1.00E+00 | 1.000 [1.000, 1.000] |
| Acquired total absence of pancreas (ICD Z90.410) | 6.17E-01 | 0.997 [0.987, 1.008] |
| Personal history of other malignant neoplasm of kidney (ICD Z85.528) | 7.03E-01 | 0.996 [0.972, 1.019] |
| ALT > 55 | 6.17E-01 | 0.995 [0.974, 1.015] |
| CA19-9 > 37 | 8.81E-01 | 0.992 [0.867, 1.118] |
| Other chronic pancreatitis (ICD K86.1) | 8.58E-01 | 0.989 [0.834, 1.144] |
| Family history of malignant neoplasm of other organs or systems (ICD Z80.8) | 8.16E-01 | 0.975 [0.405, 1.545] |
| Type 2 diabetes mellitus with hyperglycemia (ICD E11.65) | 8.16E-01 | 0.975 [0.405, 1.545] |
| Incomplete uterovaginal prolapse (ICD N81.2) | 7.88E-01 | 0.964 [0.621, 1.308] |
| Presence of urogenital implants (ICD Z96.0) | 1.88E-01 | 0.943 [0.860, 1.026] |
| Permanent atrial fibrillation (ICD I48.21) | 6.29E-01 | 0.928 [0.635, 1.222] |
| Hypertrophy of nasal turbinates (ICD J34.3) | 4.84E-01 | 0.912 [0.641, 1.182] |
| Long term (current) use of opiate analgesic (Z79.891) | 2.86E-02 | 0.898 [0.811, 0.985] |
| Body mass index (BMI) 39.0-39.9, adult (ICD Z68.39) | 4.14E-01 | 0.860 [0.525, 1.196] |
| Encounter for breast reconstruction following mastectomy (ICD Z42.1) | 6.42E-05 | 0.853 [0.786, 0.920] |
| Constipation, unspecified (ICD K59.00) | 1.51E-04 | 0.841 [0.764, 0.918] |
| Thoracic aortic aneurysm, without rupture (ICD I71.2) | 1.34E-01 | 0.841 [0.659, 1.023] |
| Chronic superficial gastritis without bleeding (ICD K29.30) | 1.02E-04 | 0.827 [0.749, 0.905] |
| Malignant neoplasm of ascending colon (ICD C18.2) | 3.17E-07 | 0.817 [0.753, 0.881] |
| Peritoneal adhesions (postprocedural) (postinfection)(ICD K66.0) | 3.28E-01 | 0.788 [0.344, 1.231] |
| Nausea (ICD R11.0) | 2.68E-01 | 0.780 [0.414, 1.145] |
| Body mass index (BMI) 37.0-37.9, adult (ICD Z68.37) | 2.22E-01 | 0.756 [0.364, 1.147] |
| Long term (current) use of insulin (ICD Z79.4) | 5.05E-02 | 0.749 [0.530, 0.969] |
| Malignant neoplasm of unspecified fallopian tube (ICD C57.00) | 1.75E-03 | 0.703 [0.546, 0.861] |
| Illness, unspecified (ICD R69) | 2.82E-03 | 0.681 [0.507, 0.856] |
| Benign lipomatous neoplasm of other sites(ICD D17.79) | 0.00E+00 | 0.671 [0.639, 0.704] |
| Encounter for surgical aftercare following surgery on the digestive system(ICD Z48.815) | 0.00E+00 | 0.636 [0.570, 0.702] |
| Other abnormal tumor markers(ICD R97.8) | 7.97E-10 | 0.627 [0.533, 0.721] |
| Acute frontal sinusitis, unspecified (ICD J01.10) | 0.00E+00 | 0.609 [0.590, 0.628] |
| Amylase < 3.4 | 1.49E-03 | 0.572 [0.389, 0.756] |
| Monoclonal gammopathy (ICD D47.2) | 1.95E-02 | 0.561 [0.263, 0.859] |
| AST > 40 | 4.08E-04 | 0.556 [0.370, 0.742] |
| Mixed hyperlipidemia (ICD E78.2) | 0.00E+00 | 0.504 [0.483, 0.525] |
| Neoplasm of uncertain behavior of digestive organ, unspecified (ICD D37.9) | 0.00E+00 | 0.478 [0.395, 0.561] |
| Overweight (ICD E66.3) | 0.00E+00 | 0.424 [0.358, 0.489] |
| Benign neoplasm of ascending colon (ICD D12.2) | 9.45E-07 | 0.375 [0.231, 0.519] |
